# Sources of electronic cigarette acquisition among school-going adolescents: A cross-sectional analysis of the 2022 National Health and Morbidity Survey – Adolescent Health Survey, Malaysia

**DOI:** 10.18332/tid/216381

**Published:** 2026-05-22

**Authors:** Kuang Hock Lim, Yoon Ling Cheong, Kuang Kuay Lim, Jia Hui Lim, Kee Chee Cheong, Sumarni Mohd Ghazali, Ali Aman Marine, Mohd Hazilas Mat Hashim, Pei Pei Heng, Chien Huey Teh, Hui Li Lim

**Affiliations:** 1 Independent Researcher, Seri Kembangan, Malaysia; 2 Institute for Medical Research, National Institutes of Health, Ministry of Health, Shah Alam, Malaysia; 3 School of Pharmacy, Faculty of Health and Medical Sciences, Taylor's University, Subang Jaya, Malaysia; 4 Biostatistics and Data Repository Sector, National Institutes of Health, Shah Alam, Malaysia; 5 Institute of Public Health, National Institute of Health, Ministry of Health, Shah Alam Malaysia; 6 Institute for Clinical Research, National Institutes of Health, Ministry of Health, Shah Alam, Malaysia

**Keywords:** electronic cigarettes, school-going adolescents, access to e-cigarettes, NHMS-AHS 2022

## Abstract

**INTRODUCTION:**

The prevalence of youth e-cigarette use has risen markedly, while traditional cigarette smoking rates have declined in recent years. Restricting access to electronic cigarettes (ECs) is regarded as a potential strategy to reduce their use among adolescents. However, there is a paucity of data on the methods by which Malaysian adolescents acquire e-cigarettes. This study seeks to identify the sources through which secondary school students in Malaysia obtain EC.

**METHODS:**

Data from the 2022 National Health and Morbidity Survey – Adolescent Health Survey, Malaysia, were analyzed. The survey employed a cross-sectional design and multi-stage sampling to obtain a representative sample of secondary school-going adolescents in Malaysia. The analysis included 4609 students who reported e-cigarette use in the past 30 days. Factors related to access and acquisition locations were examined using multivariable logistic regression and chi-squared tests.

**RESULTS:**

Among electronic cigarette (EC) users, 60.4% obtained ECs from commercial sources. Nearly 40% (39.6%) reported acquiring e-cigarettes from friends, while 36.8% purchased them from vape shops. Older age (16–17 years) (AOR=1.52; 95% CI: 1.08–2.14), male gender (AOR=1.72; 95% CI: 1.19–2.49), frequent smoking behavior (AOR=2.49; 95% CI: 1.76–3.52), and having parents who use e-cigarettes (AOR=1.71; 95% CI: 1.29–2.26) were associated with a higher likelihood of obtaining e-cigarettes from commercial sources.

**CONCLUSIONS:**

Peer-to-peer sharing was identified as the most prevalent method of e-cigarette acquisition, although a significant proportion of adolescents also purchased these products from commercial outlets. Continued research is necessary to monitor adolescent access to e-cigarettes. Interventions aimed at reducing adolescent e-cigarette use will be more effective if they address the specific sources from which these products are obtained.

## INTRODUCTION

Tobacco use patterns among Malaysian adolescents have shifted in recent years, highlighting emerging risks^1^. The prevalence of e-cigarette and other electronic vaping device use among Malaysian adolescents increased from 9.8% in 2017 to 14.9% (95% CI: 13.7–16.1) in 2022, while the prevalence of manufactured cigarette use declined from 13.7% to 6.2% (95% CI: 5.5–7.1) over the same period. In 2022, 23.5% of male adolescents reported e-cigarette use, with prevalence rising with age, from 10.7% among form one students to 19.1% among form five students. This evolving pattern necessitates consideration of new health risks, as vaping exposes adolescents to nicotine, which can harm brain development and lead to addiction^2-4^. Additionally, vaping may serve as a gateway to combustible cigarette use, with evidence indicating that e-cigarette users who initially had no intention to smoke were more likely to initiate smoking than those who reported an intention to smoke at baseline^5,6^. This suggests that vaping may be a risk factor for smoking initiation, even in the absence of other risk factors such as self-reported susceptibility^5^.

Given the widespread use of vaping and its potential risks^2-4^, understanding how adolescents access vaping devices is essential. Research shows that youth acquire tobacco products from various sources, including commercial outlets (like stores or online platforms) and social connections (such as parents, siblings, or friends)^7-11^. While much of the research has focused on how youth obtain cigarettes and other tobacco products^12,13^, there are limited data on where they get e-cigarettes^7-11^. It is important to differentiate between the sources of traditional tobacco products and e-cigarettes, as studies on tobacco access generally do not include vape shops^7,9,10^. Of the four studies on e-cigarette access, only two specifically examined vape shops, and the results varied significantly (3.8% vs 22.3% of respondents identifying vape shops as a source)^8,11^. This distinction is crucial following a recent proposal by the U.S. Food and Drug Administration (FDA) to ban the sale of flavored e-cigarettes outside vape shops and other adult-only retailers^14^. The variety of e-cigarette flavors, which are not available in traditional cigarettes, may be attractive to youth, with many studies identifying flavors as a key reason for e-cigarette use among young people; 78% of youth in one study stated that they would not use e-cigarettes if they were unflavored^15^. Understanding where youth acquire e-cigarettes, especially from vape shops, is crucial for informing regulatory decisions.

Restricting youth access to e-cigarettes, both in physical stores and online, is a potential strategy to reduce their use^16^. Recent research has demonstrated that easier access to e-cigarettes through retail outlets is associated with higher usage among youth^10^. It is important to distinguish between the acquisition of e-cigarettes and traditional tobacco products, as usage patterns differ. For instance, the social sharing and borrowing of vaping devices contrasts with the use of conventional tobacco products, indicating that adolescents may not need to own e-cigarettes to use them^11^.

To our knowledge, there has been no research on how adolescent smokers acquire e-cigarettes in Malaysia. Existing studies by Lim et al.^13,17^ have focused on cigarette procurement among adolescents based on the 2016 TECMA and NHMS 2022 studies. Findings from Western countries regarding e-cigarette sources among youth may not be generalizable to Malaysia due to cultural and social differences, variations in adolescent smoking behaviors, anti-tobacco legislation, and differing levels of enforcement regarding e-cigarette access^18-21^. Therefore, understanding how Malaysian adolescent smokers obtain e-cigarettes is essential to address this knowledge gap. Such information can help identify deficiencies in current laws and enforcement efforts aimed at reducing adolescent access to e-cigarettes. This study examines the sources of e-cigarettes and associated factors among secondary school students who use e-cigarettes in Malaysia. Identifying the sources of ECs may also serve as a valuable screening tool for clinicians or parents to detect individuals at higher risk of problematic vaping.

## METHODS

### Study design

The study employed a cross-sectional study design and a multi-stage sampling method to select a representative sample of adolescents attending secondary schools in Malaysia. This sampling was based on the 2021 secondary school framework, encompassing both private and public secondary institutions under the jurisdiction of the Ministry of Education and the Ministry of Rural and Regional Development (MARA) in Malaysia. The initial sampling phase involved stratifying Malaysia’s states and dividing each state into urban and rural categories. Proportionate-to-size sampling was then applied to identify each state’s primary sampling units (schools). Three to nine classes were chosen from each selected school through systematic random sampling, and all students in these classes were invited to participate in the study.

### Sample size and recruitment

The sample size for this study was determined using a single proportion formula to meet the objectives of each module, with the proportion derived from the previous Malaysia Global Health Survey conducted in 2017, resulting in a required sample size of 36000 after accounting for the design effect and estimating a 20% non-response rate at the national level. Consequently, 2250 adolescent respondents were needed from each state. The surveys employed an active consent approach, obtaining approval from respondents’ parents or guardians. The parents/guardians of the chosen participants received a consent form and a letter from the school administration. This letter comprehensively explained the study’s aims, emphasizing voluntary participation. The confidentiality of the respondents and the data collected was assured, with the information being used solely for research purposes. Parents or guardians who agreed to their children’s participation returned the signed consent forms to the school administration. The Adolescent Health Survey (AHS) 2022 instrument was adapted from the AHS 2017 version^22,23^. The questionnaire underwent pre-testing with a sample of students from selected primary and secondary schools in Kuala Lumpur to establish face validity and ensure the relevance of the assessment items within the local sociocultural context. Minor adjustments were made based on the feedback received during the pre-test. Data collection took place in a location designated by the school administration. No teachers or school staff were present during the data collection sessions, and only those respondents who had received approval were permitted to participate in the study. Before data collection, the research team provided a briefing to participants, outlining the survey’s objectives, the questionnaire’s content, participants’ right to decline to answer any questions, and the confidentiality of the information provided. Participants were required to sign additional consent forms.

### Variables

The dependent variable in this study was assessed by asking, ‘During the past 30 days, how many days did you use electronic cigarettes or vape?’, with the choice of: never, 1–2 days, 3–5 days, 6–9 days, 10–19 days, 20–29 days, and 30 days. The respondents who did not answer ‘never’ were further asked the question, ‘The last time you used e-cigarettes in the past 30 days, how did you obtain them?’. Respondents who indicated that they purchased e-cigarettes from a vape shop, pharmacy, online, or other commercial venues were classified as obtaining their e-cigarettes from a commercial source. Conversely, those who responded that they received e-cigarettes from a friend or family member were categorized as obtaining them from a social source. Respondents who selected ‘Other ways’ were excluded from the analysis. The independent variables examined in this study included gender, age (13–15 years, 16–17 years), ethnicity (i.e. Malay, Chinese, Indian, Bumiputra Sabah, Bumiputra Sarawak, Other), type of e-cigarette user (i.e. frequent users, defined as those who used e-cigarettes 20 days or more in the last 30 days; infrequent users, defined as those who used e-cigarettes less than 20 days in the previous 30 days)^24^, and parental e-cigarette use. All measured independent variables were considered potential confounders *a priori*.

### Statistical analysis

The dataset underwent cleaning, and the non-response rate and the research design were weighted. Descriptive statistics were used to describe the respondents’ demographic characteristics. A Rao-Scott chi-squared analysis was conducted to determine the relationship between the ‘source of e-cigarettes’ and all categorical independent variables. Variables with p≤0.25 (gender, age, parental e-cigarette use, and frequent smokers) were included in a weighted multivariable logistic regression to identify factors associated with cigarette sources from commercial sources. Additionally, all potential two-way interactions were examined to confirm the absence of any modification effects among the independent variables. All statistical analyses were performed at a 95% confidence interval (CI) (p≤0.05) utilizing SPSS statistical software version 26.

## RESULTS

The study found that 14.9% (95% CI: 13.7–16.1) of secondary school-going adolescents in Malaysia reported e-cigarette use in the past 30 days, with a substantially higher prevalence among males compared to females (79.1% vs 20.9%). Additionally, a higher prevalence of e-cigarette use was observed among respondents whose parents or guardians also used e-cigarettes (67.5%; 95% CI: 65.7–69.3). Approximately one-third of e-cigarette users were classified as frequent users (Table 1).

**Table 1:** Sociodemographic characteristics of school-going adolescents (current e-cigarette users) in the 2022 National Health and Morbidity Survey – Adolescent Health Survey, Malaysia (N=4609)

*Variable*	*Estimated population*	*Sample*	*Percent*	*95% CI*
**Gender**				
Male	241038	3559	79.1	77.0–81.1
Female	63688	1050	20.9	18.9–23.0
**Ethnicity**				
Malay	219562	3466	72.1	67.9–75.9
Chinese	18625	243	6.1	4.6–8.1
Indian	12750	165	4.2	3.0–5.8
Bumiputra Sabah	20317	311	6.7	5.3–8.3
Bumiputra Sarawak	23734	266	7.8	5.8–10.4
Other	9738	158	3.2	2.4–4.3
**Age** (years)				
13–15	164954	2351	54.1	49.5–58.7
16–17	139772	2259	45.9	41.3–50.5
**Parental marital status**				
Married	238485	3602	80.0	78.3–81.6
Separated	59738	902	20.0	18.4–21.7
**Parental e-cigarette use**				
Yes	186458	2,774	67.5	65.7–69.3
No	89685	1,395	32.5	30.7–34.3
**E-cigarette frequent user**				
Yes	187907	2,817	76.3	74.1–78.4
No	58206	880	23.7	21.6–25.9

The study revealed that 39.6% of adolescent e-cigarette users obtained their e-cigarettes from friends, while 36.8% acquired them from vape shops. Additionally, 7.8% sourced e-cigarettes from family members, 6.6% from online sources, and 6.2% from non-statutory premises (Table 2).

**Table 2:** Sources of e-cigarettes of current users among school-going adolescents in the 2022 National Health and Morbidity Survey – Adolescent Health Survey, Malaysia (N=4609)

*Source of e-cigarettes*	*Estimated population*	*Sample*	*Percent*	*95% CI*
E-cigarette shop	89000	1351	36.8	33.8–39.9
Pharmacy	6507	100	2.7	2.1–3.5
Non-static premises	15054	215	6.2	5.2–7.5
Online	16044	238	6.6	5.5–8.0
Friends	96351	1396	39.6	37.2–42.5
Family members	18890	295	7.8	6.8–9.0

The data indicate that 60.0% (95% CI: 57.0–62.9) of e-cigarette users acquired their products from commercial sources. This proportion was significantly higher among males (59.9%; 95% CI: 56.5–63.3) compared to females (44.4%; 95% CI: 33.3–47.8). E-cigarette users whose parents also used e-cigarettes were nearly 10% more likely to obtain products from commercial sources than those whose parents or guardians did not use e-cigarettes (63.2% vs 53.9%). Additionally, 73.5% of frequent e-cigarette users purchased e-cigarettes from commercial outlets, a rate nearly 1.5 times higher than that of occasional users (Table 3). Multivariable logistic regression analysis (Table 4) showed that being male (AOR=1.72; 95% CI: 1.19–2.49), frequent e-cigarette use (AOR= 2.49; 95% CI: 1.76–3.52), age 16–17 years (AOR=1.52; 95% CI: 1.08–2.14), and having parents who use e-cigarettes (AOR=1.71; 95% CI: 1.29–2.26) were associated with higher odds of obtaining e-cigarettes from commercial sources. No significant two-way interactions were detected among the independent variables (Supplementary file Figures 1–6).

**Table 3:** Source of e-cigarettes of current users by sociodemographic characteristics among school-going adolescents in the 2022 National Health and Morbidity Survey – Adolescent Health Survey, Malaysia (N=4609)

*Variable*	*Commercial source*				*Social source*				*p*
	*Estimated population*	*Sample*	*%*	*95% CI*	*Estimated population*	*Sample*	*%*	*95% CI*	
**Overall**	77449	1182	60.0	57.0–62.9	51665	768	40.0	37.1–43.0	
**Gender**									
Male	57349	863	59.9	56.5–63.3	38369	566	40.1	36.7–43.5	<0.001
Female	5680	101	40.4	33.3–47.8	8395	122	59.6	52.2–66.7	
**Ethnicity**									
Malay	44418	704	59.2	56.0–62.3	36074	49.3	40.8	37.8–44.0	0.278
Chinese	2901	42	46.2	30.1–63.1	3382	33	53.8	36.9–69.9	
Indian	2930	47	70.2	52.1–83.6	1244	13	29.8	16.4–47.9	
Bumiputra Sabah	4853	79	55.7	38.0–72.1	3852	53	44.3	27.9–62.0	
Bumiputra Sarawak	6151	65	52.6	43.6–61.5	5536	61	47.4	38.5–56.4	
Other	1773	27	46.1	29.0–64.2	2074	35	53.9	35.8–71.0	
**Age** (years)									
13–15	28302	402	51.9	46.8–57.1	26209	353	48.1	42.9–53.2	0.004
16–17	34707	562	62.8	58.1–67.3	20555	335	37.2	32.7–41.9	
**Parental use of e-cigarettes**									
Yes	23367	349	63.2	58.0–68.0	13633	198	36.8	32.0–40.6	0.005
No	32525	509	53.9	49.6–58.1	27831	414	46.1	41.9–50.4	
**Frequent e-cigarette user**									
Yes	23053	349	73.5	68.5–77.9	8327	135	26.5	22.1–31.5	<0.001
No	36212	558	51.6	47.2–56.1	33901	483	48.4	43.9–52.8	
**Parental marital status**									
Married	47149	732	56.7	52.8–60.5	33901	483	48.4	43.9–52.8	0.562
Separated	14022	201	59.0	51.9–65.7	9746	142	41.0	34.3–48.1	

Rao-Scott chi-squared was employed in the analysis.

**Table 4:** Multivariable logistic regression analysis by sociodemographic characteristics of the commercial source of cigarettes among school-going adolescents in the 2022 National Health and Morbidity Survey – Adolescent Health Survey, Malaysia (N=4609)

*Variable*	*AOR*	*95% CI*
**Gender**		
Male	1.72	1.19–2.49
Female (ref.)	1	
**Age** (years)		
13–15 (ref.)	1	
16–17	1.52	1.08–2.14
**Parents used e-cigarettes**		
Yes	1.71	1.29–2.26
No (ref.)	1	
**Frequent e-cigarette user**		
Yes	2.49	1.76–3.52
No (ref.)	1	

AOR: adjusted odds ratio.

## DISCUSSION

This national study among secondary school-going adolescents in Malaysia examined how youth obtained their e-cigarettes. The findings indicated that commercial sources accounted for the larger share of e-cigarette acquisition among current users, while social sources also contributed substantially. Peer networks were the most frequently reported social source. This finding is inconsistent with the conclusions from Gentzke et al.^25^ who reported that 32.3% of EC users obtained ECs from peers. Still, the percentage of respondents who obtained e-cigarettes from commercial sources (such as vape shops) was higher compared to the findings of several researchers who reported a lower rate, such as Baker et al.^9^ and Kong et al.^8^, who reported only around 32.5% and 38% among adolescents in New York and Connecticut in the USA. In addition, the percentage of youth getting ECs from commercial sources contradicts the findings of a scoping review conducted by Graham-DeMello et al.^26^ than noted that social sourcing was most prevalent, ranging from 43–85% (weighted mean = 75%). In five of these eight studies, social was followed by commercial sourcing, ranging from 14–41% (weighted mean = 30%). In addition, our finding is inconsistent with Do et al.^7^ who reported that the primary source of e-cigarettes was social sources (56.9%). This study also found that 6.6% obtained ECs online, although it is lower than the weighted mean of 8% reported by Graham-Demello et al.^26^. Still, it is more than 2 times higher compared to the National Youth Tobacco Survey (NYTS) study of 2. 9% of youth e-cigarette users reporting buying e-cigarettes online^25^. This higher prevalence of EC sourced from commercial sources might be due to the lack of regulation in Malaysia, which prohibits adolescents from buying e-cigarettes, compared to those in the USA, which restricts the selling of ECs to underage youth (aged ≥18 years).

The prevalence and odds of purchasing ECs from commercial sources are higher among male current EC users, with almost 60% obtaining ECs from commercial sources, compared to 40.1% among current female smokers. This finding contradicts the findings of Baker et al.^9^ and Do et al.^7^ on the method of obtaining ECs among youth in North Carolina and the USA youth, which showed a similar prevalence and odds among male and female EC users who obtained tobacco products from commercial sources. In addition, the findings also contradict the findings by Parnham et al.^27^ who reported higher odds of females obtaining ECs compared to males. The findings might be due to the difference in culture in Malaysian society, which perceives ECs as a tobacco product that is more permissive regarding smoking among male teenagers than their female counterparts. Consequently, teenage boys find it easier to obtain ECs. Research indicates that male adolescents are more inclined to source cigarettes from commercial vendors, aligning with the findings of this study. In contrast, females often exhibit reluctance to use ECs (as evidenced by their lower prevalence of EC use, 6.2% vs 23.5% among males), leading to hesitance to purchase tobacco products from commercial establishments, particularly in Malaysian and other Asian cultures that adhere to communitarian principles^13^. This philosophy prioritizes the welfare of the community and the collective good over individual desires, promoting a strong sense of community and social cohesion in which the needs of the group take precedence over personal ambitions. This observation may stem from societal tolerance, which is more permissive regarding smoking among male teenagers than female teenagers, leading to easier access to cigarettes for boys. Research indicates that male adolescents are more inclined to obtain cigarettes from commercial sources, aligning with the findings of this study^17^.

Our results show that respondents aged 15–17 years are more likely to obtain their e-cigarettes from vape shops than those in younger grades. This finding is in line with the results of the study on adolescents in the USA by Baker et al.^9^ who reported that the odds of obtaining e-cigarettes from vape shops and retail outlets are almost 2.5 times higher than those of their middle-school counterparts. Our research identified a significant positive relationship between age groups and the procurement of tobacco products from commercial establishments. This finding aligns with the results reported by Parnham et al.^27^ among youth in Great Britain, which indicated that younger individuals, particularly those aged 14–15 years, have lower odds of purchasing electronic cigarettes from supermarkets or small shops (AOR=0.45; 95% CI: 0.39–0.52; and AOR=0.52; 95% CI: 0.45–0.61) and Do et al.^7^ among youth in the USA. Concurrently, access to these products appears to decline with age. During adolescence, substantial physical and psychological changes occur, resulting in behaviors that increasingly mirror those of adults^27^; this allows older adolescents to acquire tobacco products more readily than their younger counterparts aged 13–15 years. Additionally, older teenagers generally enjoy greater independence and receive larger allowances from their parents or guardians, reflecting the increasing financial responsibilities that come with age. This age group also tends to demonstrate greater maturity and communication skills, which aid in obtaining tobacco products from commercial sources. Furthermore, older adolescents may have enhanced financial means to purchase cigarettes through commercial channels, as high school students in their later years often take on part-time jobs, enabling them to support over-the-counter purchases^13,17^.

This research indicates that current EC users whose parents or guardians smoke are more likely to obtain their ECs from commercial outlets. This observation is noteworthy, as these EC users typically prefer to obtain cigarettes from their close family members, as reported by Baker et al.^9^ among youth in the USA. Nevertheless, our results align with the outcome reported by Parnham et al.^27^ among youth in Great Britain. These findings might be due to adolescent EC users perceiving that their parents or guardians hold negative views about EC use and react to their children’s EC use behavior despite being smokers themselves. This phenomenon has been documented by Lim et al.^28,29^ in their studies involving school-aged adolescents in Kota Tinggi, Johor, and in the Petaling District, Selangor. Furthermore, Malaysian culture strongly emphasizes respecting parents, leading individuals to align with their guardians’ perceptions and behaviours^30^. Consequently, EC users may seek to maintain this respect by obtaining tobacco products that do not jeopardize familial relationships.

After adjusting for confounding variables, our study revealed that individuals who frequently use ECs are more inclined to purchase them. Similar observations have been documented in traditional cigarettes^13,17^. In addition, our findings corroborate those of Montey et al.^10^ among youth in the USA. Frequent smokers typically identify common retail locations, such as supermarkets and convenience stores, that provide easy access to ECs. Moreover, habitual smokers frequently return to the same store for their purchases, cultivating a close relationship with the store owner^13,17^, which further eases their access to cigarettes from that establishment.

### Limitations

This study is not without limitations. Though representative of students in public schools in Malaysia, the findings are applicable only to Malaysian youth; the cross-sectional study design employed in this study could not examine trends over time in access to e-cigarettes, and a causal relationship cannot be established. All results are self-reported, which may be subject to recall bias and reporting errors. In addition, such data may lead to misclassification of exposure or outcome status, potentially affecting the accuracy of the findings. In addition, although key covariates were included in the analyses, residual confounding remains, as unmeasured factors or inaccuracies in self-reported variables may have affected the findings. However, survey results are weighted to be representative of Malaysian secondary school-going adolescents. The survey did not evaluate whether young individuals obtained specific types of e-cigarettes from various locations or sources. Additionally, the survey questions and data focus on e-cigarette purchases within the past 30 days, which may not reflect the locations from which all e-cigarettes currently used by youth were acquired.

## CONCLUSIONS

The findings indicate that secondary school-going adolescents in Malaysia have relatively easy access to commercial e-cigarette sources, potentially due to insufficient restrictions on adolescent purchases. A significant majority of Malaysian youth who use electronic cigarettes (ECs) obtain them from commercial sources, with this trend being especially pronounced among frequent users, males, older adolescents, and those whose parents also use ECs. As the study is cross-sectional, causal relationships cannot be established. Nevertheless, the results highlight several areas for future research, including the evaluation of public health campaigns and community-based interventions targeting adolescent e-cigarette use. The effects of recent Malaysian government regulations, such as the 2024 prohibition of sales to individuals aged <18 years, also warrant investigation, particularly regarding enforcement strategies and their impact on youth access. Further research could explore the effectiveness of regulatory measures such as e-cigarette outlet licensing, age-verification protocols, and monitoring of marketing practices in limiting adolescent access.

## Supplementary Material



## Data Availability

The data supporting this research are available from the authors on reasonable request.
