# Supplementary material for: Sources of electronic cigarette acquisition among school-going adolescents: A cross-sectional analysis of the 2022 National Health and Morbidity Survey – Adolescent Health Survey, Malaysia
Source: Tob Induc Dis. 2026 May 22;24:10.18332/tid/216381. doi: 10.18332/tid/216381 (PMC13197885; doi:10.18332/tid/216381)
Supplement: Supplementary file 1 [file TID-24-63-s1.pdf]

Supplementary file

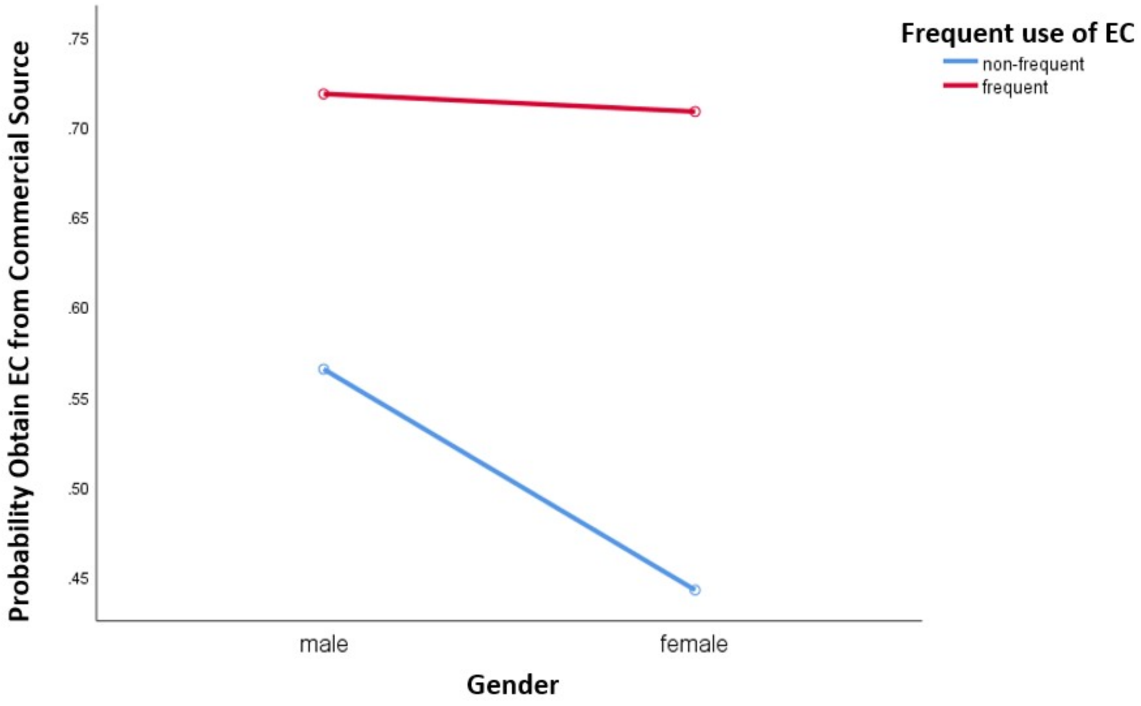

Figure 1. Interaction analysis between gender and frequent e-cigarette (EC) use among secondary school-going adolescents who participated in the 2022 National Health and Morbidity Survey – Adolescent Health Survey, Malaysia (N=4609)

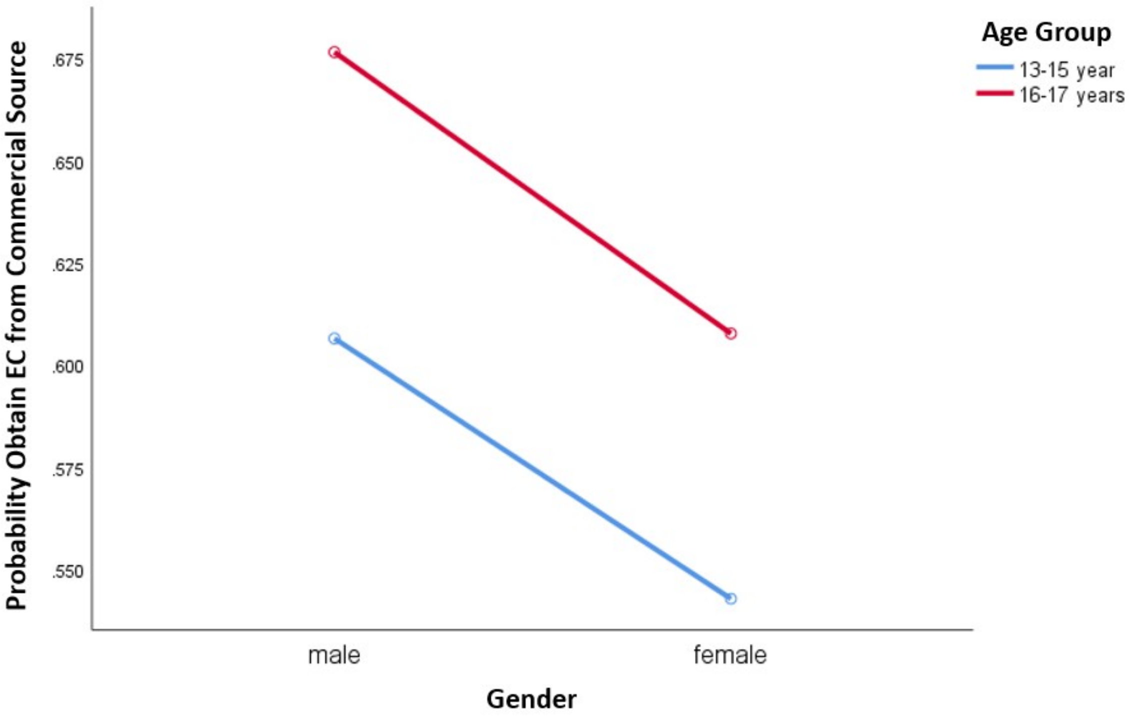

Figure 2. Interaction analysis between gender and age group among secondary school-going adolescents who used e-cigarettes (ECs) in the 2022 National Health and Morbidity Survey – Adolescent Health Survey, Malaysia (N=4609)

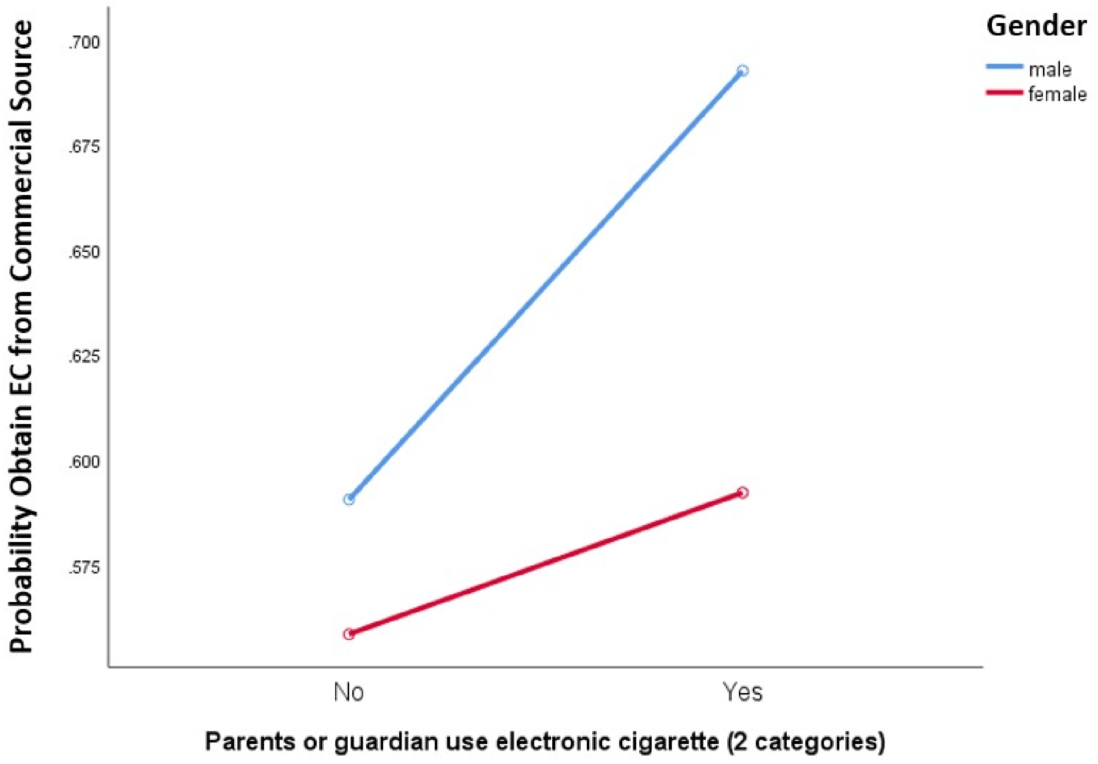

Figure 3. Interaction analysis between gender and parents or guardians' use of electronic cigarettes (ECs) among secondary school-going adolescents who used ECs in the 2022 National Health and Morbidity Survey – Adolescent Health Survey, Malaysia (N=4609)

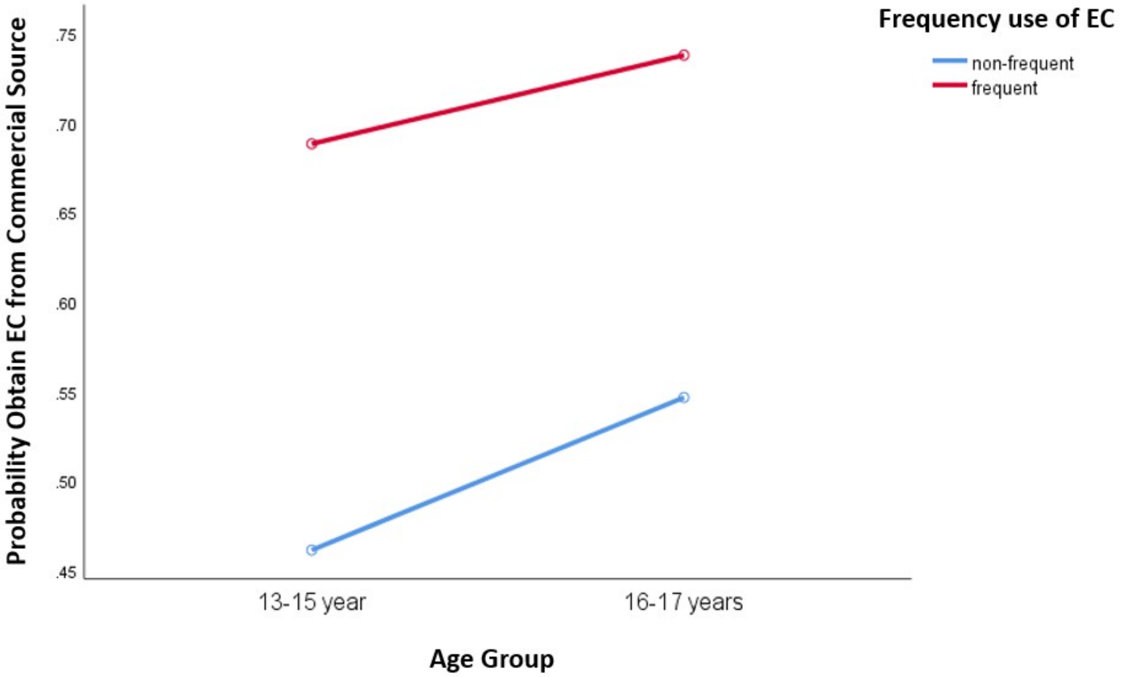

Figure 4. Interaction analysis between frequent e-cigarette (EC) users and age group among secondary school-going adolescents who used ECs in the 2022 National Health and Morbidity Survey – Adolescent Health Survey, Malaysia (N=4609)

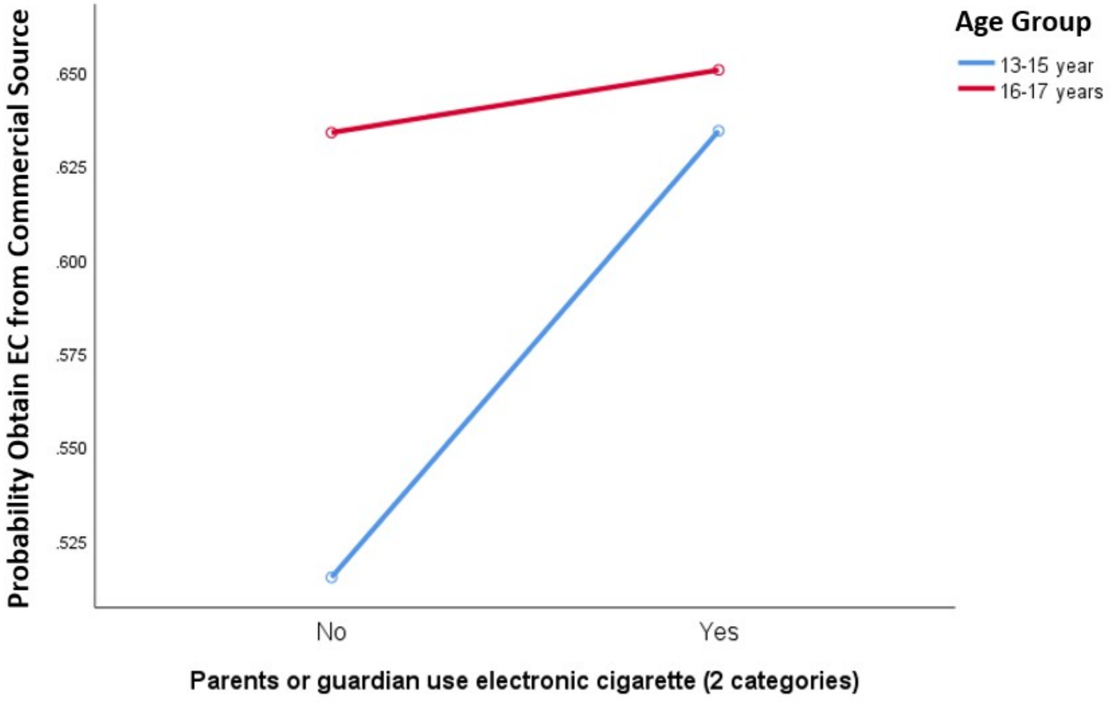

Figure 5. Interaction analysis between parents or guardians who use e-cigarettes (ECs) and age group among secondary school-going adolescents who used ECs in the 2022 National Health and Morbidity Survey – Adolescent Health Survey, Malaysia (N=4609)

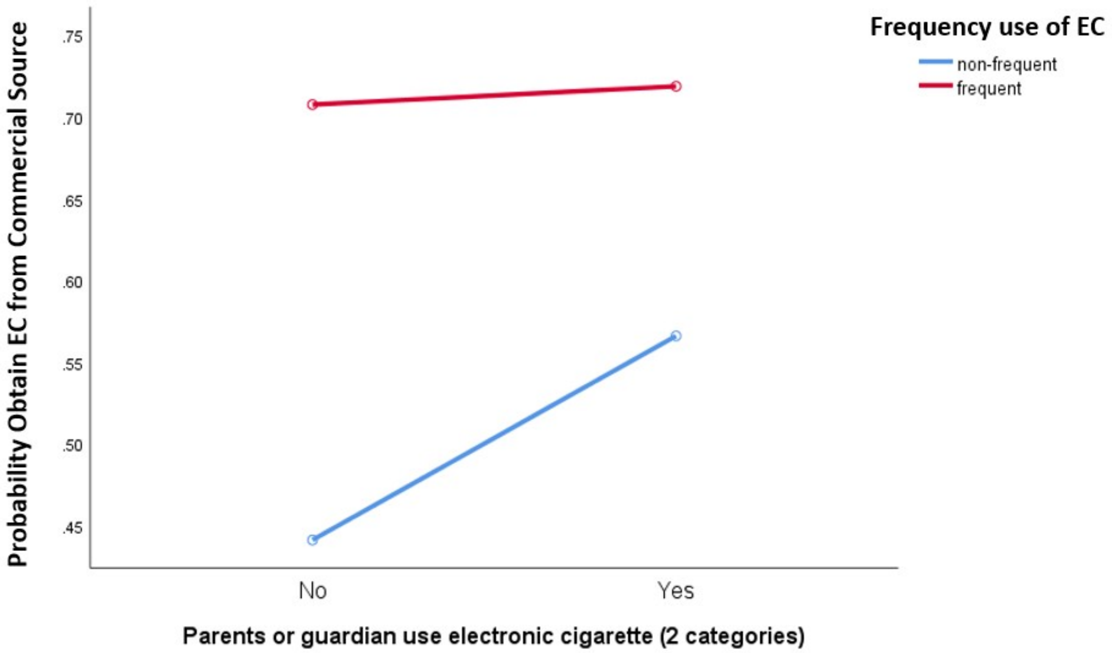

Figure 6. Interaction analysis between frequent e-cigarette (EC) users and parents or guardians who use ECs among secondary school-going adolescents who used ECs in the 2022 National Health and Morbidity Survey – Adolescent Health Survey, Malaysia (N=4609)

© 2026 Lim KH. et al.
